# Enteric hyperoxaluria in chronic pancreatitis

**DOI:** 10.1097/MD.0000000000006758

**Published:** 2017-05-12

**Authors:** Nathalie Demoulin, Zaina Issa, Ralph Crott, Johann Morelle, Etienne Danse, Pierre Wallemacq, Michel Jadoul, Pierre H. Deprez

**Affiliations:** a Division of Nephrology, Cliniques universitaires Saint-Luc; b Institut de Recherche Expérimentale et Clinique; c Institut de Recherche Santé et Société, Université catholique de Louvain; d Department of Radiology; e Department of Clinical Chemistry; f Department of Hepato-Gastroenterology, Cliniques universitaires Saint-Luc, Brussels, Belgium.

**Keywords:** kidney disease, malabsorption, oxalate nephropathy, steatorrhea

## Abstract

Chronic pancreatitis may lead to steatorrhea, enteric hyperoxaluria, and kidney damage. However, the prevalence and determinants of hyperoxaluria in chronic pancreatitis patients as well as its association with renal function decline have not been investigated.

We performed an observational study. Urine oxalate to creatinine ratio was assessed on 2 independent random urine samples in consecutive adult patients with chronic pancreatitis followed at the outpatient clinic from March 1 to October 31, 2012. Baseline characteristics and annual estimated glomerular filtration rate (eGFR) change during follow-up were compared between patients with hyper- and normo-oxaluria.

A total of 48 patients with chronic pancreatitis were included. The etiology of the disease was toxic (52%), idiopathic (27%), obstructive (11%), autoimmune (6%), or genetic (4%). Hyperoxaluria (defined as urine oxalate to creatinine ratio >32 mg/g) was found in 23% of patients. Multivariate regression analysis identified clinical steatorrhea, high fecal acid steatocrit, and pancreatic atrophy as independent predictors of hyperoxaluria. Taken together, a combination of clinical steatorrhea, steatocrit level >31%, and pancreatic atrophy was associated with a positive predictive value of 100% for hyperoxaluria. On the contrary, none of the patients with a fecal elastase-1 level >100 μg/g had hyperoxaluria. Longitudinal evolution of eGFR was available in 71% of the patients, with a mean follow-up of 904 days. After adjustment for established determinants of renal function decline (gender, diabetes, bicarbonate level, baseline eGFR, and proteinuria), a urine oxalate to creatinine ratio >32 mg/g was associated with a higher risk of eGFR decline.

Hyperoxaluria is highly prevalent in patients with chronic pancreatitis and associated with faster decline in renal function. A high urine oxalate to creatinine ratio in patients with chronic pancreatitis is best predicted by clinical steatorrhea, a high acid steatocrit, and pancreatic atrophy. Further studies will need to investigate the mechanisms of renal damage in chronic pancreatitis and the potential benefits of therapies reducing oxaluria.

## 1 Introduction

Chronic pancreatitis (CP) describes a wide spectrum of fibroinflammatory disorders of the pancreas that eventually lead to failure of exocrine and endocrine pancreatic functions.^[1]^ It results from alcohol abuse, cigarette smoking, genetic mutations, autoimmune, and obstructive disorders. The main symptoms of CP are intermittent or persistent abdominal pain, exocrine pancreatic insufficiency, and diabetes. Unfortunately, treatment largely remains empirical, and many patients require interventional procedures and frequent hospital admissions.^[2–5]^

Pancreatic exocrine dysfunction has been anecdotally associated with hyperoxaluria both in native and transplanted kidneys.^[6,7]^ Mechanisms of hyperoxaluria in CP include calcium binding by fatty acids, increased intestinal absorption of uncomplexed oxalate, and increased colonic permeability to oxalate.^[8–11]^ Enteric oxaluria may in turn lead to oxalate nephropathy through crystal deposition in the renal parenchyma. However, the prevalence and determinants of hyperoxaluria and its association with renal function decline are unknown among patients with CP.

We performed an observational study and show that hyperoxaluria is present in nearly a quarter of patients with CP and associated with markers of pancreatic exocrine dysfunction and with renal function decline.

## 2 Methods

### 2.1 Study design

All adult patients with CP seen at the gastroenterology outpatient clinic in Cliniques universitaires Saint-Luc in Brussels were invited to participate in this study from March 1 to October 31, 2012, and prospectively handled.

The diagnosis of CP was based on the presence of at least 1 clinical criterion (episodic or persistent pain, attacks of acute pancreatitis, diabetes mellitus, and steatorrhea) or well-defined complication (bile duct, duodenal or vascular obstruction/stenosis with clinical signs, pancreatic fistula, ascites, or pseudocysts) together with abnormality in pancreas imaging (ductal irregularity or dilatation, parenchymal changes including general or focal enlargement of the gland, cysts, calcifications, and parenchymal atrophy), according to the recent classification of CP.^[12]^ Steatorrhea was defined as the demonstration of fat malabsorption with an excretion of >6 g of fat/day in a 72-hours stool collection or a fecal acid steatocrit >31%.^[13]^ Diabetes mellitus was ascertained on the basis of either antidiabetic drug treatment or biochemical evidence of diabetes.^[14]^ Pancreatic atrophy was defined as a pancreatic size <15 mm.^[15–18]^

All patients were interviewed and examined by the same gastroenterologist (senior author). Alcohol abuse was defined as regular alcohol intake of more than 50 g/day in men and 25 g/day in women.^[19,20]^ Clinical steatorrhea was defined by more than 3 loose, pale and/or floating stools.^[21]^ Estimated glomerular filtration rate (eGFR) was calculated using the Chronic Kidney Disease Epidemiology Collaboration creatinine equation.^[22]^ Fecal acid steatocrit and elastase-1 levels were measured in all patients, using a semiquantititave measure (separation of acidified stool suspension into 3 solid, aqueous, and lipid layers, with measure of the latter) and enzyme-linked-immunosorbent serologic assay kit (Bioserv Diagnostics GmbH, D-17489 Grieswald, Germany), respectively. Oxaluria was measured twice using an enzymatic assay on acidified random urine spot sample (Trinity Biotech Oxalate Kit, Jamestown, NY) and reported as urinary oxalate to creatinine ratio (UOCR). Patients were classified as hyperoxaluric if UOCR was >32 mg/g.^[23]^ eGFR was monitored during follow-up.

The study was approved by the Ethics committee of the Faculty of Medicine of Université Catholique de Louvain (B403201213280). Written informed consent was obtained from all participants.

### 2.2 Statistical analysis

Data are shown as the mean ± SD. A comparison between 2 UOCR measures in every patient was made using a paired *t*-test. The Student *t*-test was used to compare continuous variables and *z*-test for proportions. Binary dependent and continuous dependent variables were analyzed by multivariate logistic and ordinary least squares regression analyses, respectively. *P*-values <.05 were considered as statistically significant.

The mean annual change in eGFR during follow-up was calculated for every patient from 2 to 4 successive eGFR assessments obtained at 12-month intervals.

All analyses were performed using GraphPad Prism (version 6.01) or Stata (version 12) software.

## 3 Results

### 3.1 Patient characteristics

Characteristics of the 48 enrolled patients are provided in Tables 1 and 2. The mean age was 58 years and the sex ratio was 1:1. Etiology of CP was toxic (alcohol or smoking) in 52%, idiopathic in 27%, obstructive in 11%, autoimmune in 6%, and genetic in 4% of patients. The diagnosis of CP had been made 7.2 ± 7.4 years earlier. Half the patients were on pancreatic enzyme supplementation and 17% had clinical steatorrhea. The mean eGFR was 89 ± 21 mL/min/1.76 m^2^ and urinary protein to creatinine ratio (UPCR) 0.1 ± 0.3 g/g. Fecal acid steatocrit level was >31% in 52% of patients, and fecal elastase level was decreased in 60% (<200 μg/g).

**Table 1:**
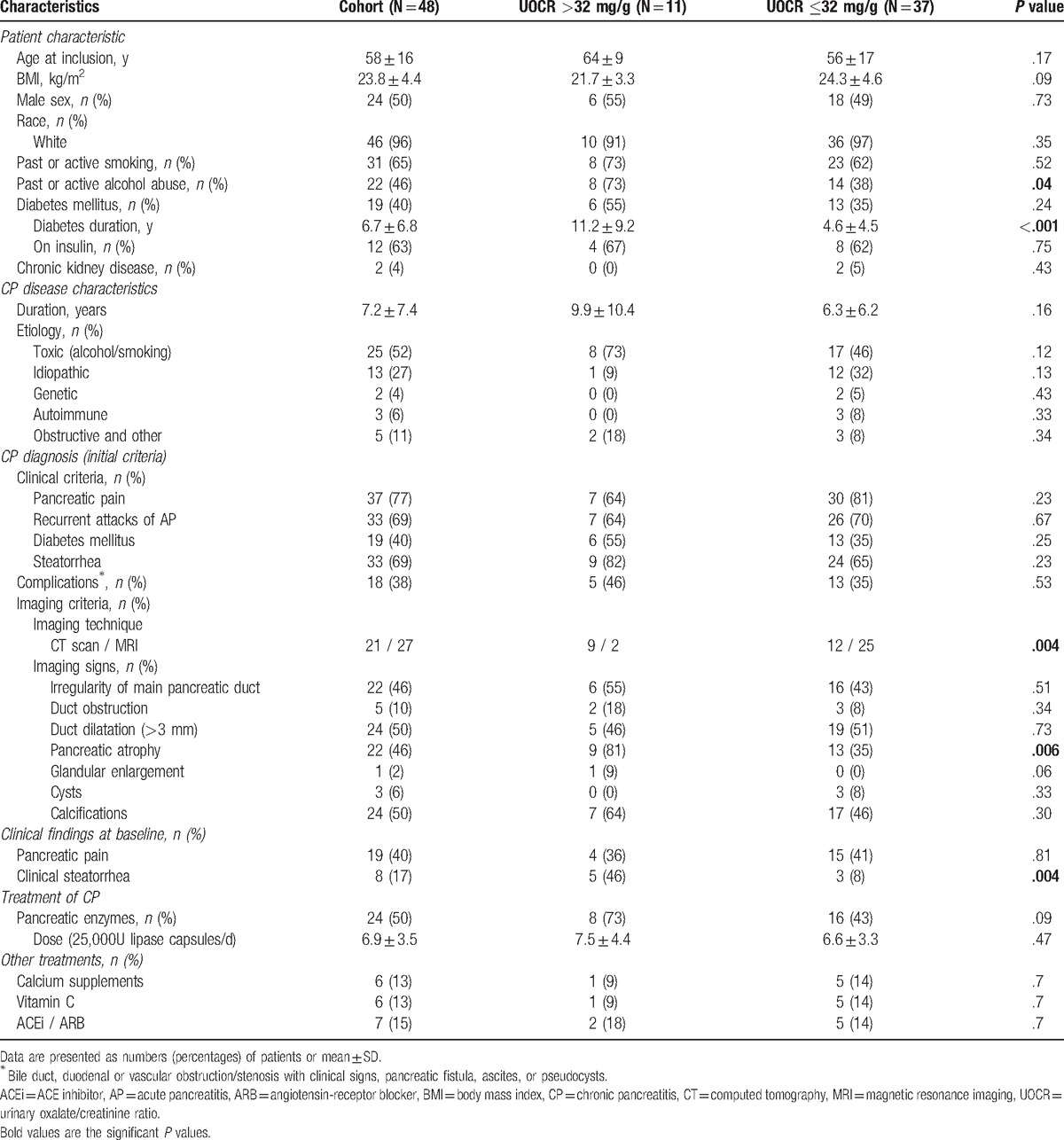
Baseline demographic and clinical characteristics.

**Table 2:**
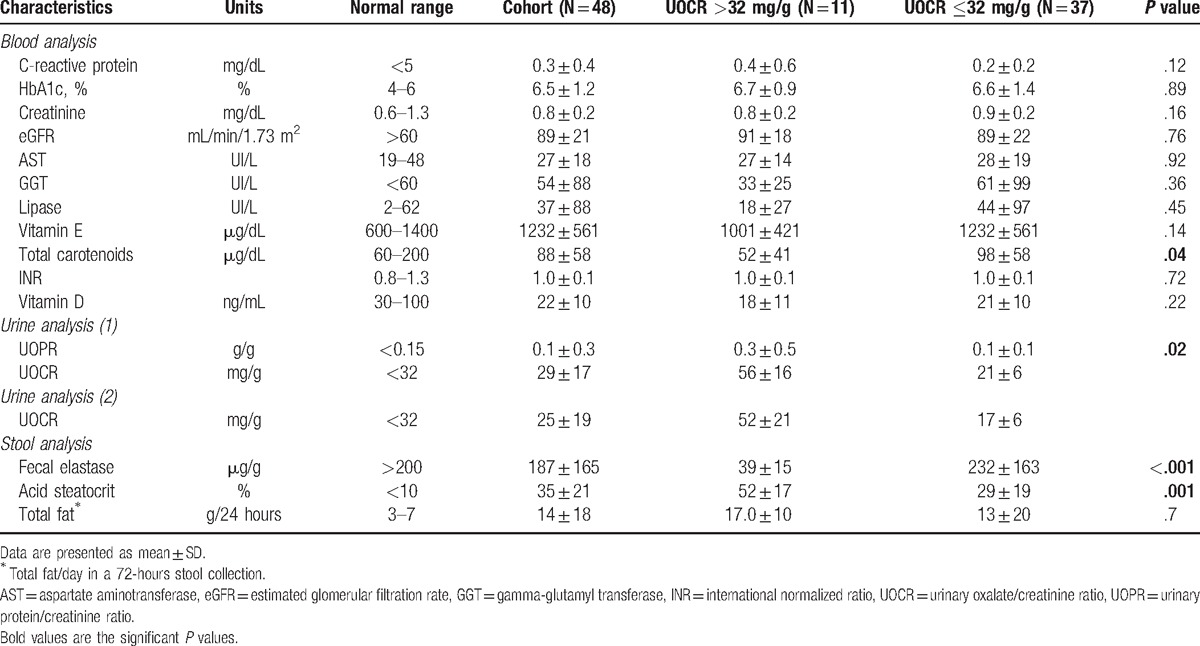
Baseline biological data.

### 3.2 Prevalence and determinants of hyperoxaluria

High levels of UOCR (>32 mg/g) were present in 23% of the patients. An intermeasure agreement between both urine samples was excellent: all normo-oxaluric patients had a normal second oxaluria measure and all hyperoxaluric patients except one again were hyperoxaluric.

Comparison of clinical and biological variables in hyperoxaluric and normo-oxaluric patients showed that pancreatic atrophy (*P* = .006), clinical steatorrhea at inclusion (*P* = .004), excessive alcohol intake (*P* = .04), and long-term diabetes (*P* <.001) were clinical characteristics more frequent in patients with UOCR >32 mg/g. These patients had a longer duration of disease (9.9 vs 6.3 years), and were more frequently on pancreatic enzymes (73 vs 43%) when compared to normo-oxaluric patients, although these differences were not significant. Among the biological findings, low carotenoids level (*P* = .04), higher UPCR (*P* = .02), low fecal elastase-1 level (*P* <.001), and high fecal acid steatocrit (*P* = .001) were associated with hyperoxaluria (Tables 1 and 2).

**Figure 1:**
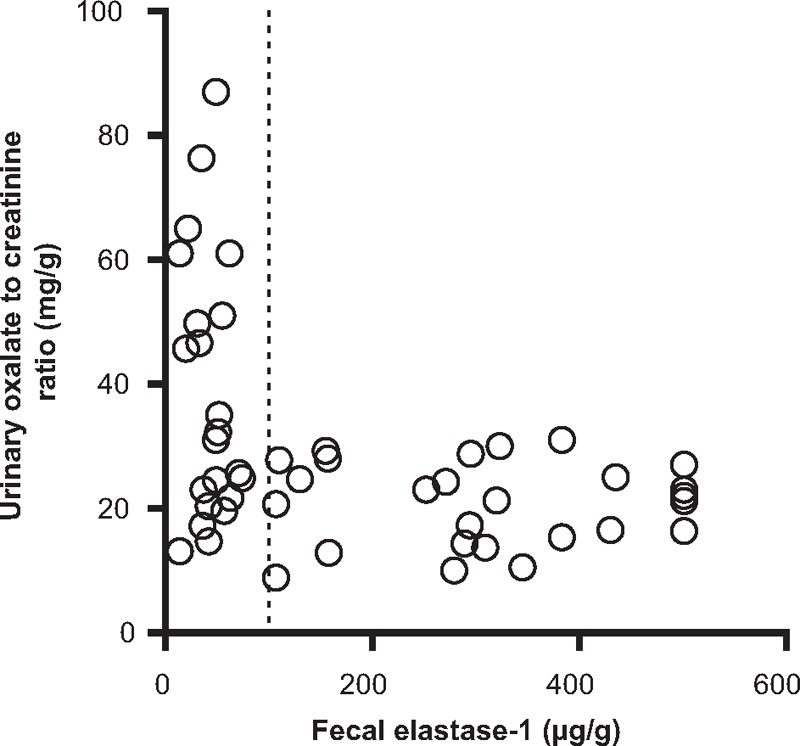
All patients with fecal elastase-1 measure >100 μg/g (at the right of the dotted line) had a normal urinary oxalate/creatinine ratio (<32 mg/g).

The distribution of UOCR as a function of fecal elastase levels is shown in Fig. 1. All 26 patients with elastase levels >100 μg/g were normo-oxaluric.

The regression analysis explained 52.7% of the variance of the oxaluria measures with only clinical steatorrhea, high steatocrit level, and pancreatic atrophy as significant predictive variables (Table 3). Inclusion of “pancreatic enzyme therapy” as a binary covariate in the regression analysis, after having removed the nonsignificant covariates, did not modify these associations (data not show). The presence of clinical steatorrhea, steatocrit level >31%, and pancreatic atrophy was associated with a positive predictive value of 100% and a negative predictive value of 86% of having hyperoxaluria.

**Table 3:**
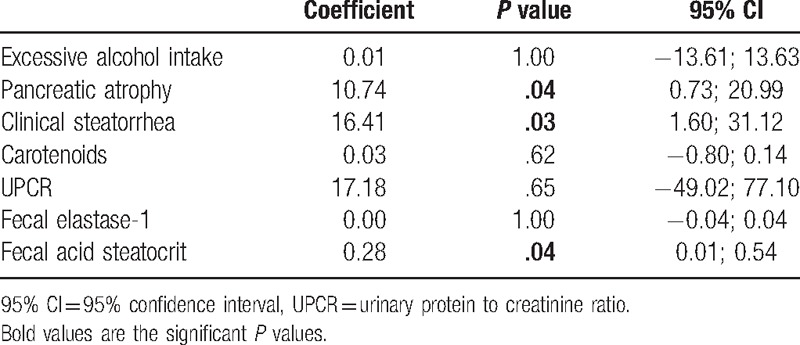
Multivariate analysis of the factors associated with hyperoxaluria.

Altogether, these data show a high prevalence of hyperoxaluria in patients with CP and a significant association with markers of exocrine dysfunction.

### 3.3 Comparison of mean annual eGFR change in patients with hyperoxaluria versus normo-oxaluria

Follow-up data on renal function were available in 7/11 (64%) patients with UOCR >32 mg/g and in 27/37 (73%) patients with normo-oxaluria at baseline. The mean duration of follow-up was 904 days, with no difference between patients with or without hyperoxaluria (*P* = .59). The mean annual decline in eGFR was highly variable in this limited number of patients with longitudinal follow-up, and values of –6.4 ± 3.4 and –0.1 ± 1.9 mL/min/1.73 m^2^ were observed in patients with UOCR >32 mg/g or ≤32 mg/g, respectively (*P* = .10). Annual change in eGFR closely correlated with the baseline level of UOCR (Spearman, *r* = 0.39, *P* = .02). UOCR >32 mg/g was significantly and independently associated with a higher risk of eGFR loss during follow-up after adjustment for established determinants of renal function decline, including gender, diabetes, bicarbonate level, baseline eGFR, and UPCR (Table 4).

**Table 4:**
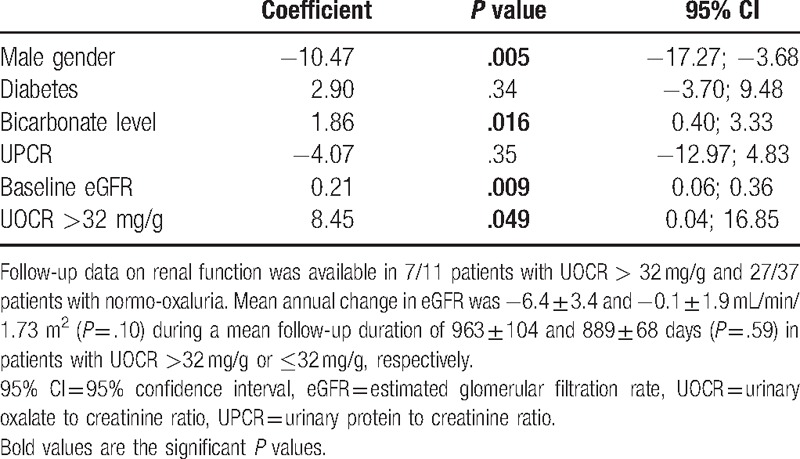
Multivariate analysis of the factors associated with renal function decline.

## 4 Discussion

Our study shows for the first time the high prevalence of hyperoxaluria among prevalent patients with CP seen at the outpatient clinic, with almost a quarter of them affected. Enteric hyperoxaluria has also been reported in other malabsorptive disorders such as chronic inflammatory bowel disease, short bowel syndrome, and bariatric surgery.^[24–27]^ In comparison, hyperoxaluria was present in about half of patients following modern bariatric surgery.^[25]^

In our cohort, clinical steatorrhea, a high fecal acid steatocrit and pancreatic atrophy were found to be independent predictors of high UOCR. Steatorrhea is a feature of advanced stages of CP and does not usually occur until pancreatic lipase output drops below 10% to 15% of normal levels.^[1]^ The relationship between pancreatic exocrine dysfunction and increased oxaluria was also well illustrated by the fact that all patients with elastase level >100 μg/g had normal oxaluria values. Past studies have also shown a strong correlation between steatorrhea and urinary oxalate excretion in patients suffering from other malabsorptive states.^[26,28]^

We also found a baseline UOCR >32 mg/g to be associated with mean annual eGFR decline rate during follow-up, although this needs to interpreted with caution because of the limited number of patients and the high inter-individual variability in eGFR changes. The clinical expression of hyperoxaluria is extremely variable and depends on its severity, chronicity, etiology, and on the presence of other factors.^[10,29]^ Hyperoxaluria leads to urinary calcium oxalate supersaturation, and eventually the formation and retention of calcium oxalate crystals. This may result in diffuse renal calcifications (nephrocalcinosis) or stones (nephrolithiasis).^[10,30–31]^ Also, calcium oxalate crystal deposits in renal tubules may induce epithelial lesions and tubular necrosis, and crystals can migrate into the interstitium triggering inflammation.^[10,32]^ This “acute oxalate nephropathy” has been reported in patients with inflammatory bowel disease, celiac disease, jejunoileal and gastric bypass, CP, gastrointestinal lipase inhibitor use, and pancreatic carcinoma.^[6,24,33–35]^ Previously impaired renal function, hypovolemia, and poor adherence to pancreatic enzymatic substitution treatment are suspected to increase the risk of developing oxalate nephropathy in patients with CP and hyperoxaluria.^[36–37]^ Further large-scale, prospective studies will be needed to properly examine the impact of high urinary oxalate levels on long-term kidney function in patients with CP.

We used spot urine samples normalized to creatinine to determine oxaluria. This has recently emerged as a reliable and easy alternative to 24-hour urine oxalate quantification, much alike the now universally adopted urinary albumin to creatinine ratio in the context of chronic kidney disease.^[38–40]^ Studies have shown the absence of significant diurnal pattern of UOCR.^[39,41]^ Additionally, there was a good agreement between the 2 oxaluria measures in our patients.

The limitations of our study are the inclusion of prevalent CP patients, half of them already under treatment. This should intuitively have limited the extent of steatorrhea, thereby limiting the oxaluria. Thus, our results may be an underestimate of the actual prevalence of hyperoxaluria in CP. Although 72-hour fecal fat excretion is still considered as the gold standard for the diagnosis of fat malabsorption, its use is limited in the clinical setting. Several studies have shown acid steatocrit performed on a random stool sample to have a good sensitivity and specificity for the detection of steatorrhea.^[13,42,43]^ Another drawback is the absence of restrictive dietary measures adopted during urine collection. However, Stauffer^[26]^ found urinary oxalate to be not significantly different in normal subjects on controlled diets versus those ingesting a regular diet with added tea. Moreover, we showed a good reproducibility of the oxaluria levels in our patients.

Altogether, we prospectively demonstrated a high (around 25%) prevalence of hyperoxaluria in a selected cohort of CP patients. Hyperoxaluria is associated with clinical and biological signs of intestinal malabsorption and exocrine pancreatic insufficiency, and possibly with eGFR change during a mean follow-up of 2.5 years. Further studies are needed to determine the clinical significance of hyperoxaluria in patients with CP and identify those most likely to benefit from treatment in order to prevent renal complications, including dietary counseling, pancreatic enzyme replacement therapy, use of oxalate binders, and sufficient hydration.
